# Transplantation in paediatric patients with MMA requires multidisciplinary approach for achievement of good clinical outcomes

**DOI:** 10.1007/s00467-023-05906-0

**Published:** 2023-02-25

**Authors:** Alicia Paessler, Miriam Cortes-Cerisuelo, Wayel Jassem, Hector Vilca-Melendez, Akash Deep, Vandana Jain, Andrew Pool, Stephanie Grunewald, Nicos Kessaris, Jelena Stojanovic

**Affiliations:** 1 Great Ormond Street Hospital for Children NHS Foundation Trust, Great Ormond Street, WC1N 3JH, London, UK; 2 University College London Great Ormond Street Institute of Child Health, London, UK; 3 King’s College Hospital NHS Foundation Trust, London, UK; 4 Guys and St Thomas’ NHS Foundation Trust, London, UK

**Keywords:** Paediatrics, Kidney transplantation, Liver transplantation, Abdominal wall transplantation, Methylmalonic acidaemia, Williams syndrome

## Abstract

**Background:**

As modern medicine is advancing, younger, small, and more complex children are becoming multi-organ transplant candidates. This brings up new challenges in all aspects of their care.

**Methods:**

We describe the first report of a small child receiving a simultaneous liver and kidney transplant and abdominal rectus sheath fascia transplant on the background of Williams syndrome and methylmalonic acidaemia. At the time of transplantation, the child was 3 years old, weighed 14.0 kg, had chronic kidney disease stage V, and had not yet started any other form of kidney replacement therapy.

**Results:**

There were many anaesthetic, medical, metabolic, and surgical challenges to consider in this case. A long general anaesthetic time increased the risk of cardiac complications and metabolic decompensation. Additionally, the small size of the patient and the organ size mis-match meant that primary abdominal closure was not possible. The patient’s recovery was further complicated by sepsis, transient CNI toxicity, and de novo DSAs.

**Conclusions:**

Through a multidisciplinary approach between 9 specialties in 4 hospitals across England and Wales, and detailed pre-operative planning, a good outcome was achieved for this child. An hour by hour management protocol was drafted to facilitate transplant and included five domains: 1. management at the time of organ offer; 2. before the admission; 3. at admission and before theatre time; 4. intra-operative management; and 5. post-operative management in the first 24 h. Importantly, gaining a clear and in depth understanding of the metabolic state of the patient pre- and peri-operatively was crucial in avoiding metabolic decompensation. Furthermore, an abdominal rectus sheath fascia transplant was required to achieve abdominal closure, which to our knowledge, had never been done before for this indication. Using our experience of this complex case, as well as our experience in transplanting other children with MMA, and through a literature review, we propose a new perioperative management pathway for this complex cohort of transplant recipients.

**Supplementary Information:**

The online version contains supplementary material available at 10.1007/s00467-023-05906-0.

## Introduction

As modern medicine has advanced and the medical management of complex health conditions is improving, more children with previously fatal complex medical backgrounds are surviving. This means that younger and smaller children with metabolic conditions and children requiring multi-organ transplantation are increasingly becoming transplant candidates in the twenty-first century. This new cohort of transplant patients brings up new challenges not just for paediatricians and transplant physicians, but also for healthcare professionals caring for these patients as they transition into adulthood. Here we describe the first reported case of a 3-year-old child receiving a combined liver and kidney transplant (CLKT) and a subsequent abdominal rectus sheath fascia transplant on the background of two rare conditions: Williams syndrome (WS) and methylmalonic acidaemia (MMA). This case has flagged a range of new anaesthetic, metabolic, transplant, and surgical challenges which required a multidisciplinary approach and from which many lessons were learned. In addition, we review the literature and our own practice of transplanting children with MMA and propose a peri-operative management pathway for this complex cohort of transplant recipients. In our centre, to date, 2 patients underwent CLKT (both from deceased donors), 2 had liver only (both from deceased donors), and 2 had kidney only transplants (both from living donors).

MMA is an autosomal recessive disorder that results in a deficiency of methylmalonyl-coenzyme A mutase leading to the accumulation of methylmalonic acid and other metabolites which leads to metabolic acidosis, metabolic stroke, poor feeding, pancreatitis, cardiomyopathy, developmental delay, and kidney tubular damage ultimately leading to kidney failure [1]. Due to the frequent progression to kidney failure, it is suggested that kidney transplantation (KT) should be considered in the management of children with MMA [2]. However, liver transplantation (LT) is also sometimes carried out with the aim of supplying the deficient enzyme and therefore reducing the sequelae associated with MMA. There are reports of patients receiving isolated LT [3–5] or KT [6, 7], as well as CLKT [7–10] with no clear consensus on which is the optimum approach. A recent multi-centre study found that plasma MMA levels and eGFR at 2 years post-transplant had significantly improved in patients receiving LT or CLKT, compared to patients receiving a KT [11]. This study attributed these differences to higher enzymatic activity being provided by the transplanted liver. These findings were mirrored by another study looking at a smaller French cohort [9] as well a published literature review [12], suggesting that LT and/or CLKT should also be considered in the management of children with MMA when other medical options have been exhausted. The literature also suggests that patients with KT or LKT have fewer episodes of post-transplant metabolic decompensations [13–15]. Another study reported long-term outcomes in their cohort of transplanted MMA patients and found that following CLKT neurodevelopment stabilised and plasma MMA levels significantly decreased and so they recommended to opt for CLKT in patients where CKD is present [8]. However, as with any patient, the benefits of organ transplantation need to be balanced with their immediate and long-term risks. Due to the complexities of these patients and the risks associated with organ transplantation, their transplantation should occur at centres with good availability of senior clinicians with experience with these conditions, working closely with metabolic teams to reduce post-operative complications and mortality [12].

Williams syndrome (WS) is a multi-system disorder that occurs as a result of a deletion on chromosome 7q11.23. The most common findings in a person with WS are cardiovascular anomalies which are the leading cause of morbidity and mortality in affected patients. Patients typically also have a degree of global cognitive impairment, abnormal arterial vasculature, short stature, hypercalcaemia, glucose intolerance and failure to thrive. Due to the cardiovascular anomalies, a small proportion of patients experience sudden cardiac death, which is particularly pronounced during general anaesthesia and requires careful risk stratification. The management of patients with WS is largely focused on correction of the cardiovascular issues but requires a multidisciplinary approach to manage all the affected organ systems as well as their neurodevelopmental profile [16].

## Case

A male patient carried to full term was genetically diagnosed in the neonatal period with both WS and MMA after presenting with hyperammonemia at 7 h of life which prompted further testing, as neither of these conditions are routinely screened for in newborns in the UK. The patient’s genetic cause of the MMA was confirmed to be due to mutase deficiency (mut0). Additionally, the patient had an antenatal diagnosis of a multicystic dysplastic kidney. The patient was born to non-consanguineous parents who had two other healthy children and no family history of any abnormalities, although they had one pregnancy which was terminated due to the foetus showing several severe abnormalities of the genitourinary tract.

As a result of WS the child had several symptomatic hypercalcaemic episodes during infancy, requiring hospitalisation and treatment with pamidronate on multiple occasions. He also was found to have three ventricular septal defects (VSD) and pulmonary stenosis. Unfortunately, the patient’s MMA was not responsive to hydroxocobalamin therapy and so by 2 years of age the patient showed a rapid deterioration in his kidney function which progressed to Chronic Kidney Disease (CKD) Stage V. This was a result of tubulointerstitial disease of MMA on a background of kidney dysplasia. The patient underwent an extensive assessment of his metabolic, transplant and cardiovascular status in order to gain a better understanding of his complex health needs to formulate a long-term management plan. Some guidelines recommend transplantation before patients have any significant neurological events and when they are under stable metabolic conditions [17], which was the current situation for this patient at the time of assessment. Following multiple multidisciplinary meetings, discussions at the regional MMA meeting, discussion with the family and based on the current evidence [9, 11, 12] it was felt the best option for this patient was a CLKT as he had reached CKD Stage V and the addition of the liver would provide better long-term outcomes. As no living donors were available for a sequential LT then KT, the patient was assessed for listing on a deceased donor waiting list for CLKT. Prior to listing the patient, an extensive protocol was made for the pre-, peri- and post-operative period in anticipation of the many possible challenges, the most important being small patient size and the need for two solid organ intra-abdominal transplants and metabolic decompensation during long general anaesthetic. In addition, as the patient was on the waiting list for a deceased donor organ, meaning that organs can become available at any time (such as overnight or during the weekend when there are generally reduced services), proposed protocol had to incorporate detailed and clear management plan which would be user friendly. This protocol can be found as Supplementary File 1. Preparation for surgery required ongoing collaboration between hepatologists, nephrologists, kidney transplant surgeons, liver transplant surgeons, metabolic teams, cardiologists, dieticians, intensivists and anaesthetists.

At the time of organ offer, the patient had MMA levels of 2,837,902 umol/mL, serum creatinine of 267 umol/L, eGFR of 12.4 ml/min/1.73 m^2^ (eGFR measured with iohexol, as is the standard for MMA patients at our centre) with a height of 91.5 cm and a weight of 14.0 kg. At the time of the organ offer, the patient’s MMA was being managed with carnitine. The patient had not yet started dialysis, although prior to organ offer, conversations had been held regarding the approaching need for dialysis. Following discussions with the MDT, although peritoneal dialysis might have helped to expand the abdominal cavity in preparation for post-transplant abdominal closure, the family decided that if the need for dialysis were to arise prior to transplantation, they would personally prefer to opt for haemodialysis. However, the organ offer was made prior to the commencement of any type of dialysis. Some evidence suggests that the use of intra-operative or pre-operative haemodialysis can reduce intra-operative death and this is routine in some centres [18]. However, we do not routinely use dialysis intra-operatively for MMA patients, and none of our MMA patients received pre-operative haemodialysis and thus far this has not led to any peri-operative metabolic complications. Other centres may want to consider pre-operative haemodialysis in MMA patients that are already established on dialysis prior to transplant.

Prior to transplantation, the patient had not suffered any metabolic decompensations, episodes of pancreatitis, or strokes; however, the patient did have some global developmental delay which was attributed to a combination of continuous hyperammonaemia and WS. While there was a size mis-match between the donor and the patient it was felt that accepting this organ and ensuring transplantation happened while the patient was metabolically stable, before suffering any metabolic decompensations and before the patient required dialysis, would provide better long-term outcomes based on current evidence in the literature. Additionally, a larger liver from a larger donor also provides greater enzymatic activity and so aids in reducing plasma MMA levels [17].

## Anaesthetic challenges

One aspect that was anticipated to be challenging with this patient was the long period under general anaesthetic. Although rare, there are reports of sudden death during general anaesthesia in children with WS so it was important that this was carefully planned. While currently there are no trials or clear guidelines on anaesthetic management of these patients, a careful analysis of their cardiovascular issues at one month prior to the procedure is key to establish the patient risk and plan for any potential challenges [19]. Additionally, an ECG should be obtained shortly before any anaesthesia as there is growing evidence that a long QTc on ECG puts a patient at higher risk of sudden cardiac death [16]. For our patient, ECHO confirmed closed VSDs with mild pulmonary stenosis and normal cardiac function (fractional shortening > 30%). These findings put him in a medium risk category. In view of known effects of MMA on cardiac function, six monthly ECHOs should be done for all patients with MMA.

Another potential anaesthetic risk was the risk of hypercalcaemia and the consequences that could result, e.g. arrhythmias and seizures. Hypercalcaemic episodes are common in children with WS, and while this patient’s hypercalcaemic episodes appeared to have stabilised since infancy, it was still an important risk to consider and plan for [20].

With increasing time under general anaesthesia, there is also an increase in risk of metabolic decompensation in MMA patients. While our patient did not have any metabolic decompensations both prior to and peri-transplant, he required long general anaesthetic, with anticipated operating time being 10 h, therefore putting him in a high risk for metabolic decompensation. This patient had previous general anaesthetic with no complications but only for a brief period of time (less than 60 min for the gastrostomy insertion). A CLKT would include significant physiological stress, an expected anaesthetic time of 10–12 h, and a risk of sepsis in the post-operative period, so the chances of metabolic decompensation peri- and post-operatively were significant. Meticulous attention is required to avoid hypothermia, coagulopathy and maintain glycaemic control, all of which can be challenging during liver transplantation. Additionally, it was important to avoid post-operative nausea and vomiting as this could cause dehydration and acidosis and further increase the risk of metabolic decompensation [21]. While the majority of the anaesthetic management (pre-operative ECG, ECHO, avoiding hypercalcaemia, hypothermia and glycaemic control) of this patient is standard as with all patients undergoing transplantation, due to the devastating consequences metabolic decompensation can have, including death, it was important to follow this care meticulously and to have a plan put in place to monitor for any metabolic changes and how these would be addressed once they occurred.

In this patient, the anaesthetic aspect of the transplant ran smoothly with a total of 9 h of general anaesthesia. There were periods of hypotension post-reperfusion due to bleeding and hypovolaemia although this was managed with red cell transfusion and use of colloid-based intravenous fluids.

## Metabolic challenges

The overarching metabolic goal when managing this patient was to avoid metabolic decompensation peri-transplant. A key factor to this was to adequately prepare for any catabolism that was likely to occur.

Nutrition is a constant challenge in MMA patients and was even more complicated in this patient as he required a bespoke feed that was low in calcium, restricted protein intake, yet also addressed this patient’s failure to thrive. Typically, patients are required to be nil by mouth prior to transplantation which would lead to catabolism. Therefore, with the expert input from the metabolic team and the dieticians, and taking into consideration fluid volume requirement in view of kidney failure, at the time of the organ offer, this patient was started on his emergency feed regimen via his gastrostomy up until 6 h prior to the anaesthetic induction. The emergency feed was then changed to IV fluids and IV dextrose throughout the pre- and peri-operative period which was given as recommended in the literature [12]. To avoid protein catabolism and metabolic decompensation this fluid regimen was continued for the first 24 h post-transplant. Protein was re-started 24 h after transplant and was guided by ammonia levels. The patient continued on bespoke total parenteral nutrition as per the transplant protocol until trophic feeds were started on day 12 post-transplant. As a consequence of the continued use of dextrose in this patient, hyperglycaemia was an additional challenge that was overcome with an insulin sliding scale. The patient had 4 hourly blood gasses pre-surgery to ensure metabolic stability which increased to half hourly arterial blood gasses analysis during surgery and hourly post-surgery and for the first 24 h.

Based on the literature [12] and our experience, we recommend detailed assessment of the patient’s metabolic state from the time of admission with baseline blood tests including venous blood gas, ammonia and lactate to ensure the patient is not in a catabolic state or in active metabolic decompensation prior to transplant. This monitoring should continue 4 hourly until surgery. Any evidence of metabolic instability should warrant further assessment by the kidney and liver transplant teams. Surgery should be delayed until metabolic stability is achieved. While a longer cold ischaemic time for transplanted organs can lead to poorer outcomes in transplantation [22], having a good understanding of a complex patient’s metabolic state helps inform the management of the peri- and post-operative course. For full metabolic assessment, we propose the following investigations: venous blood gas, U&Es, LFTs, clotting, FBC, cross match, glucose, lactate, ammonia, ketones, amylase, lipase, urate, plasma amino acids, homocysteine, acylcarnitine profile, plasma, and urinary MMA, FGF21 (due to new evidence suggesting it is a highly predictive marker of metabolic stress in patients with MMA [23, 24]), troponin and PTH.

As anticipated, long general anaesthetic and major surgery in a patient with a metabolic condition led to the patient having the highest lactate of 14 mmol/L on admission to PICU post-transplant although this later decreased and was between 3 and 7 mmol/L which was deemed acceptable and also was within the patient’s baseline. As a result of the detailed protocol and preoperative planning that were put in place for this patient, he did not have any metabolic decompensations peri-transplant. The positive effect of the transplant was soon noticed in the significantly improving MMA levels which decreased from 2,837,902 umol/mL pre-transplant to 369,250 umol/mL and 100,139 umol/mL on days 4 and 45 post-transplant, respectively. At 6 months post-transplant MMA levels were found to be 177 umol/mL. While these levels are still above the normal range, they have shown a significant improvement that would not have been possible without the transplant and have had an immense clinical benefit to the patient. Patient growth and neurodevelopmental status improved as well at the last follow-up 18 months after the transplant.

## Medical challenges

During the early post-operative course (first 28 days), there were a number of medical complications including two episodes of sepsis, tacrolimus toxicity, and de novo DSAs. Day 2 post-op, the patient’s respiratory function deteriorated, and he required high frequency ventilation. Blood cultures were negative with *Candida albicans* found in the peritoneal fluid. The patient recovered well from this episode; however, at 1-month post-op he became pyrexial, had a rising CRP and grew *Pseudomonas* in his urine. On the suspicion of urosepsis the ureteric stent was removed as well as the urinary catheter and the patient soon recovered.

On day 25 post-transplant, the patient had a transient AKI, and so became oliguric and his creatinine doubled from 40 to 80 umol/L and then rose to 96 umol/L within a 12-h period. Initially, antibody mediated rejection (ABMR) was suspected as at this point the patient also developed de-novo DSAs against HLA-DQAI*01:03(5009), DQAI*05:05(8392 l) with MFI 13,401 so the patient was pulsed with high-dose methylprednisolone. Up until this point, in terms of immunosuppression, the patient was induced with 10 mg of basiliximab on Day 0 and Day 4 as well as 600 mg/m^2^ of methylprednisolone intra-operatively and then maintained with daily tacrolimus and prednisolone with tacrolimus levels being 16–21 ng/mL (target tacrolimus level was 10–12 ng/ml for this time post-transplant). Additionally, mycophenolate was introduced from day 4 post-transplant at a dose of 600 mg/m^2^ twice daily for 14 days, then reduced to 300 mg/m^2^ twice daily. Kidney transplant biopsy confirmed diagnosis of tacrolimus-induced acute tubular necrosis with no evidence of ABMR. Therefore, on day 26, tacrolimus was weaned, and the new target created for levels to be in the range 5–7 ng/mL. Following pulsed methylprednisolone, DSAs then showed a continuous reduction of MFI to 1,758 with a cRF of 73% at 6 months post-transplant. At the last follow-up 18 months post-transplant, DSAs are negative and patient maintenance immunosuppression consists of tacrolimus, MMF, and prednisolone.

## Surgical challenges

The main surgical challenge encountered in this patient was his small size at the time of transplant. Both at the time of listing and time of transplantation, the patient was below the 0.4^th^ centile for height and weight, weighing 14 kg at time of transplant. His small stature and low weight were thought to be a consequence of his kidney failure as well as his WS. Small size carried additional risks including thrombosis and anastomotic leak, but these did not occur in this patient nor in any of other patients in our cohort. There are also reported challenges with abdominal closure in patients < 15 kg following any abdominal transplantation in which there is an organ size mismatch [25]. It has been reported that patients who have end stage liver disease with associated hepatomegaly and ascites, or patients receiving peritoneal dialysis, have an expanded abdominal cavity and therefore have fewer difficulties with abdominal closure [26]. A commonly used approach when there is a size mismatch in organs is to have a delayed abdominal closure once any oedema associated with the initial transplant surgery has reduced at 7–10 days post-transplant [25]. Another approach to this issue that is well-documented is not only to transplant a split segment of the whole liver, but also to reduce the size of this segment further until it is of adequate size [27].

In this case, the patient did not have end stage liver disease and therefore had no ascites, nor was he on peritoneal dialysis so did not benefit from an expanded abdominal cavity.

The patient underwent a CLKT from a 36-year-old DBD with a 221 HLA mismatch and CMV mismatch (donor CMV IgG positive, recipient CMV IgG negative). He received a reduced left liver lobe with the reduction being performed ex situ resulting in a graft recipient weight ratio of 3.7. The transplant technique included caval replacement, conventional arterial anastomosis between the common hepatic artery of donor and recipient, and duct to duct anastomosis for the biliary tree. The cold ischaemia time for the liver was 8 h and 46 min, while the anastomotic time was 43 min. The histology of the explanted native liver showed mild focal steatosis, nuclear glycogen, and grade 3 siderosis.

After the liver was transplanted, the right colon was mobilised to expose the lower part of the IVC and abdominal aorta. Careful retraction was performed throughout the procedure so that the bowel and liver perfusion were not affected inadvertently. Once the vessels were dissected adequately for kidney transplantation, vascular clamps were applied on the IVC first to anastomose the renal vein, using 5/0 prolene and then onto the aorta to anastomose the renal artery, using 6/0 prolene. The clamps were then released, and kidney perfusion assessed to be satisfactory, before performing Lich-Gregoir ureteric anastomosis onto the bladder, using 4/0 PDS, over a double-J ureteric stent. The cold ischaemia time for the kidney was 11 h and 27 min with an anastomotic time of 40 min.

At the end of the surgery due to the space occupied by the new organs and also to avoid compression syndrome, abdominal wall was left open; therefore, a silastic mesh fixed to the edges of the muscle was used as a method of temporary abdominal closure.

On day 11 post-transplant, abdominal closure was attempted; however, this was unsuccessful and so the abdomen was once again covered with silastic mesh. At this point, multidisciplinary discussions ensued with a decision to use a donor rectus sheath fascia to achieve abdominal closure.

There are multiple types of abdominal wall transplants, each with their own risks and benefits. For this patient, the most suitable was a non-vascularized, non-composite abdominal rectus muscle fascia graft. This was because the patient had insufficient fascia to close the abdomen but did have enough skin that could be mobilized to achieve skin-to-skin closure if enough fascia was in place [25]. There have been fewer than 20 reported cases of this procedure in paediatric patients, none of which were following CLKT so this was a novel approach. The most common uses for this type of abdominal wall transplant are following multivisceral or liver and intestinal transplantation [28].

**Fig. 1:**
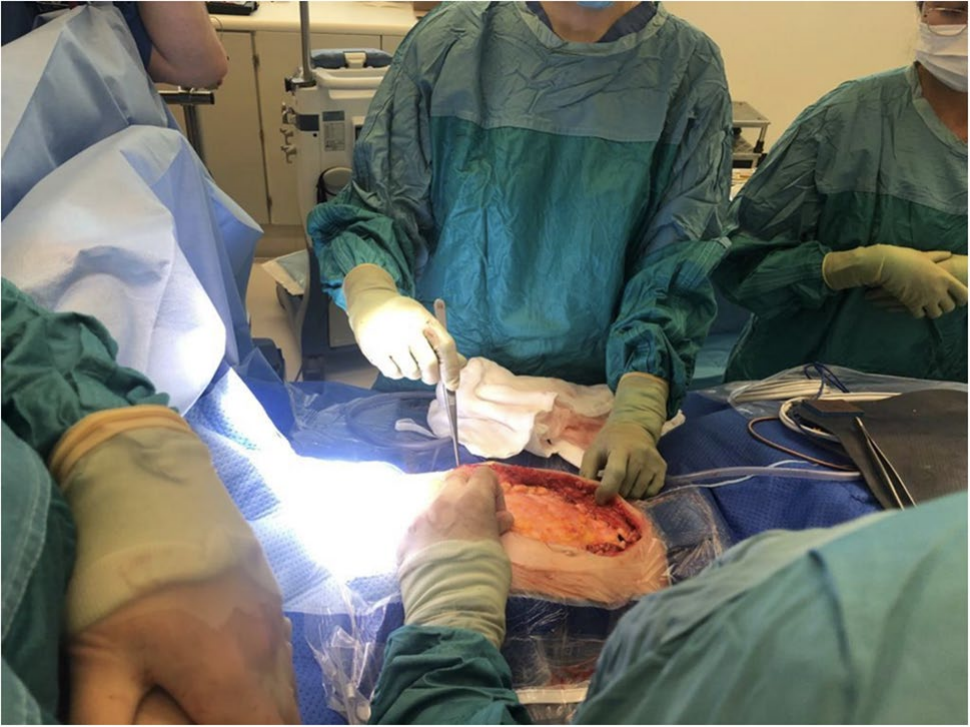
Donor rectus fascia placed in the patient’s abdomen

**Fig. 2:**
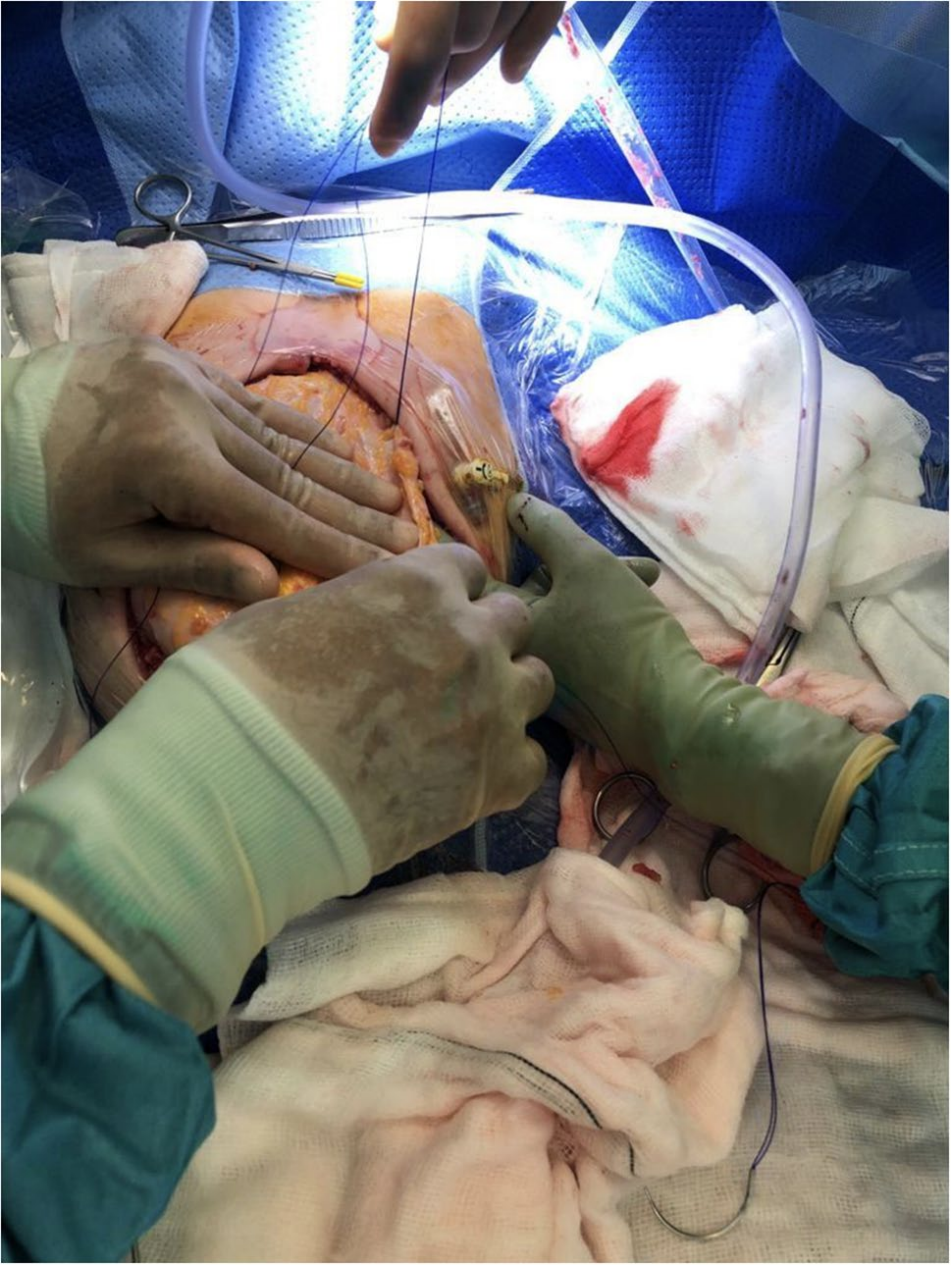
Fixing of the donor fascia to the patient’s abdominal muscle

After a period of stability, the patient was put on a national list to receive a compatible blood group rectus muscle fascia from a deceased donor for the definitive closure of the abdominal wall defect. Forty days post-transplant, a rectus muscle fascia from a 37-year-old DBD became available. Once retrieved, and after removing the donor rectus muscle on the back table as described elsewhere [25], the fascia was fixed to the edges of the patient’s abdominal muscle with absorbable suture as seen in Figs. 1 and 2. The skin was closed covering the fascia with interrupted stitches.

Eleven days following this procedure, the patient developed a subcutaneous collection which required drainage but showed no growth on aspirate. Additionally, the patient developed abdominal wall cellulitis which cleared with a 5-day course of IV antibiotics. Both are recognised complications of fascia transplants and fortunately were both managed well with no anticipated long-term consequences. At 2 months post-transplant, the patient was able to be discharged home.

Currently at 18 months post-transplant, the patient is doing very well, has shown good growth in weight and height (both now on the 25^th^ centile), and has shown excellent improvement in developmental milestones with neurodevelopment growth in gross and fine motor skills with improving social milestones. He has stable kidney and liver function, significantly reduced MMA levels, has not required any further interventions and his abdomen has healed well. Some studies suggest that patients should continue on protein restriction post-transplant due to the ongoing release of muscle MMA [12]; however, following slow incremental titration, this patient is tolerating the maximum protein intake recommended for the healthy population of his age (1.28 g/kg/day) with stable plasma MMA levels and kidney function with close monitoring. We recommend protein restriction post-transplant is managed on an individualized basis. He is also no longer vomiting and is increasing his oral intake of food.

Using this approach to transplantation with our other MMA patients has led to a 5-year patient survival rate of 85% with one patient death at 3 years post-kidney transplant of acute heart failure. Death censored allograft survival remains 100%.

## Summary of recommendations

**Fig. 3:**
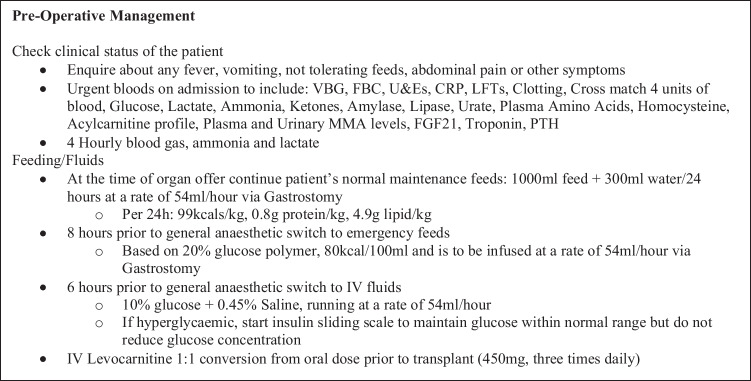
Medical and metabolic pre-operative management protocol used in this patient. Infusion rates and volumes of feeds and fluids are based on the patient’s usual tolerance. VBG, venous blood gas; FBC, full blood count; U&Es, urea & electrolytes; CRP, C-reactive protein; LFTs, liver function tests; FGF21, fibroblast growth factor 21; PTH, parathyroid hormone; IV, intravenous

**Fig. 4:**
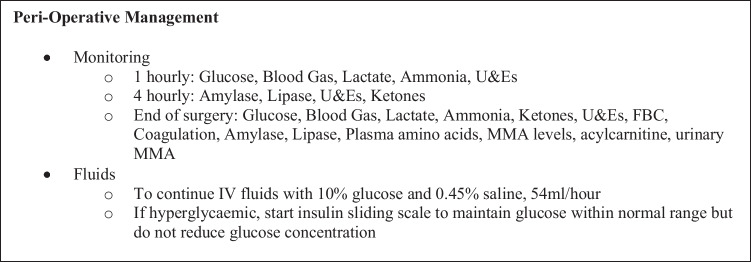
Medical and metabolic peri-operative management protocol used in this patient. infusion rates and volumes are based on the patient’s usual tolerance

**Fig. 5:**
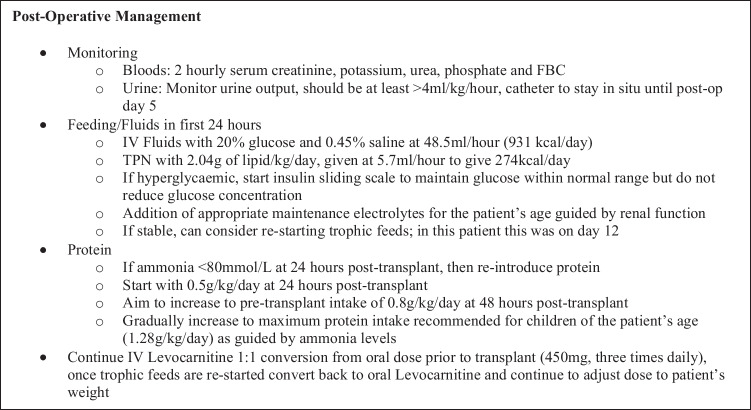
Medical and metabolic post-operative management protocol used in this patient. Infusion rates and volumes of feeds and fluids are based on the patient’s usual tolerance. TPN, total parenteral nutrition

Living donation should be the preferred choice of transplantation due to the multiple benefits including optimisation of metabolic status pre-operation and longevity of organs coming from living vs. deceased donors being some of them. It is important to develop patient-specific individualized management protocols for the pre-, peri-, and post-operative period that consider the patient’s management prior to transplant. The specific guidelines used at our institution for this patient regarding their medical and metabolic management can be seen in Figs. 3, 4, and 5. A more generalised and patient non-specific template for transplant protocol can be seen in Supplementary File 1.

A proposed management plan for patients with a metabolic condition, such as MMA, undergoing transplantation should include the following:

1. Involvement of multi-disciplinary teams from the time of transplant assessment onward with essential professionals being anaesthetists, hepatologists, nephrologists, intensivists, cardiologists, metabolic clinicians, kidney and liver transplant surgeons and dieticians with expertise in metabolic and kidney conditions.
2. Consideration for emergency feeds to start at the time of the organ offer being accepted.
3. Clinical assessment to include full set of metabolic bloods on admission (venous blood gas, U&Es, LFTs, clotting, FBC, cross match, glucose, lactate, ammonia, ketones, amylase, lipase, urate, plasma amino acids, homocysteine, acylcarnitine profile, plasma and urinary MMA, FGF21, troponin and PTH). Any biochemical evidence of metabolic instability should prompt further investigations by transplant and metabolic teams. A patient who has evidence of metabolic instability should not undergo surgery until their metabolic status improves.
4. If the patient has metabolic stability on admission, venous blood gasses should be checked at least every 4 h.
5. Hourly venous blood gasses should be done intraoperatively and at least once hourly in the first 24 h post-surgery.
6. Replacement fluid should be based on metabolic requirement and for the remaining fluid that is often needed for kidney allograft perfusion, consideration should be given to crystalloid fluids with no added glucose or colloid fluids.
7. Availability of senior metabolic, kidney and liver transplant physicians should be ensured for the pre- and peri-operative period including the first 48 h post-surgery.
8. A holistic approach in post-operative management should continue as multidisciplinary approach with joint liver and kidney transplant reviews.
9. Immunosuppression should be personalised for each patient and we refrain from advising on that aspect in this paper. We report good clinical outcomes in our patients with basiliximab being used as induction agent, and tacrolimus, MMF and steroids as maintenance immunosuppression.
10. Transplantation of children with MMA should be considered and done in centres experienced in doing complex paediatric transplants with well-established kidney and liver transplant and metabolic services.

## Conclusion

In conclusion, we have presented the case of a 3-year-old child who underwent combined liver and kidney transplantation followed by abdominal rectus sheath fascia transplantation on a background of methylmalonic acidaemia and Williams syndrome. This is the first reported case of a patient with this background undergoing combined solid organ transplantation and also the first reported case of an abdominal wall transplant being used to achieve abdominal closure following CLKT. Despite this patient’s complex medical background and the complications encountered post-operatively, this patient has achieved excellent outcomes which would not have been possible prior to transplantation. Many lessons were learned from this case which will undoubtedly improve the management of future patients undergoing multi-organ transplantation with complex medical backgrounds. This case has shown that it is essential to have tailored and detailed protocols in place prior to transplantation. Transplant teams caring for patients with metabolic conditions may also consider gaining an in depth understanding of the patient’s metabolic status at the time of transplantation, even if it causes delays and prolongs cold ischaemic time. Additionally, we have shown that abdominal rectus sheath fascia transplants can be a good solution for achieving abdominal closure in small patients receiving transplants with a size mismatch. Most importantly, a multidisciplinary approach is key when caring for such patients and for the achievement of good clinical outcomes.

## Supplementary Information

Below is the link to the electronic supplementary material.

Supplementary file1 (DOCX 27 KB)

## Data Availability

De-identified participant data are available upon reasonable request from JS at jelena.stojanovic@doctors.org.uk.

## Declarations

### Disclaimer

The views expressed are those of the authors and not necessarily of the NHS, the individual hospital trusts or the Department of Health.
