# Supplementary material for: Transplantation in paediatric patients with MMA requires multidisciplinary approach for achievement of good clinical outcomes
Source: Pediatr Nephrol. 2023 Feb 25;38(8):2887–96. doi: 10.1007/s00467-023-05906-0 (PMC10393894; doi:10.1007/s00467-023-05906-0)
Supplement: Supplementary file 1 — Supplementary file1 (DOCX 27 KB) [file 467_2023_5906_MOESM1_ESM.docx]

**(Name of Transplant Centre)**

***Transplant Protocol for patient X (weight X kg)***

*******When patient is called for a simultaneous liver-kidney transplant:*******

***When accepting organs and BEFORE starting the surgery***

1. Hepatobilliary team to discuss with the Renal Transplant Surgeon on call
2. Hepatobiliary Team to inform Paediatric Nephrologist on call
3. *Renal transplant surgeon to discuss with the HLA Tissue Typing Lab* ***Patient will require a HLA cross-match result to be available prior to kidney implantation.***
4. *Liver and renal surgeons to discuss surgical approach depending on donor size / split liver etc.*

**Protocol contents:**

1. **Demographics and information about patient daily care/requirements and potential complications and risks**
2. **Management at the time when organs are accepted and before admission**
3. **Management on admission and before the patient goes to theatre**
4. **Intraoperative management**
5. **Management post operation in the first 24 hours**
6. **Key metabolic and renal contacts during and our of hours**
7. **Additional**

**1. Demographics and information about X’s daily care/requirements and potential complications and risks**

| **Name:** |  |
| --- | --- |
| **D.O.B:** |  |
| **Diagnosis:** |  |
| **Dialysis Modality:** |  |
| **Transplant Type:** |  |
| **Previous transplants** |  |
| **Date Activated on Waiting List for Deceased Donor Organ:** |  |

Does patient have central venous access?

**(Potential) metabolic risks for this patient:**

Prolonged Fasting, Catabolism

(Lactic) acidosis

Pancreatitis – Consider using SMOF when on TPN

Encephalopathy

Metabolic stroke

Cardiomyopathy, arrhythmias

Hyperglycaemia

Hypoglycaemia (cave: sensitive to insulin)

Hyperfiltration / thrombosis

Hyperhydration / hypertension

**Usual Maintenance gastrostomy feeds: (please note patient does not tolerate more than Xml volume / 24h)**

(Insert gastrostomy feed ingredients)

*Per 100ml: X kcal, Xg protein, Xg fat, X% CHO*

*Per 24 hours: X kcal (X kcals/kg), X g protein (Xg/kg)*

Volume/24hours: X ml feed plus X ml water flushes

Feed given as boluses: 07.30 X ml, 10.00 X ml, 11.30 X ml, 14.00 X ml, 15.30 X ml, 17.00 X ml, 19.30 X ml, 22.00 X ml, 23.30 X ml

**Emergency feeds (based on 20% glucose polymer):**

Maximum volume tolerated usually (X ml/hour)

*Per 100ml: X kcal In total volume of X ml: X kcal*

**IV fluids (when fasted):**

Glucose 10% / 0.45% sodium chloride continuous infusion at constant rate to give Maximum tolerated volume / 24 hours

1. **Management at the time when organs are accepted and before admission**

| **Task** |  | **Done by** |
| --- | --- | --- |
| When called for transplant, check clinical status of the patient (fever, vomiting, symptoms of any illness) | If any concerns, discuss with on call transplant and metabolic teams | Admitting team |
| Feed/fluids plan | - Start patient on their normal maintenance feeds (as above) as continuous feed via Gastrostomy at (maximum tolerated volume usually)/hour at the time of organ offer - Change to emergency feeds (as above) via gastrostomy 8 hours prior to general anaesthetics |  |
| If vomiting | Discuss with on call metabolic team | Admitting team |

1. **Management on admission and before the patient goes to theatre**

| **Task** |  | **Done by** |
| --- | --- | --- |
| A recipient serum sample for DSA assay is to be taken at the time of admission in all SLK  cases, to enable prospective and retrospective cross-matching. | Send sample urgently to Clinical transplantation | Admitting team |
| If the recipient has no DSAs and the HLA cross match is negative, then the kidney transplant can proceed with standard SLK  immunosuppression (see below) and no special considerations. |  | Admitting team to chase and discuss with renal consultant and renal transplant surgeon **before** surgery |
| Clinical examination (specific questions relevant for metabolics ? tolerating feeds, ? vomiting, ? abdominal pain) | If concerns, inform liver and renal transplant surgeons as well as nephrologist and metabolic consultant on call.  Patient is considered to be clinically unwell if:   - abnormal blood gas with ph < 7.3 and / or HCO3 < 16 and/or BE > - 4 and / or lactate > 4mmol/l - and / or ammonia > 100nmol - and / or amylase and / or lipase 2 x above normal - LFT 2 x above normal | Admitting team |
| Bloods to be sent urgently | Blood gas, U&E, CRP, LFTs, clotting, FBC, cross match 4 units of blood, glucose, lactate, blood gas, ammonia, ketones, amylase and lipase, urate, plasma amino acids, homocysteine, acylcarnitine profile, plasma and urinary MMA, FGF21, troponin, PTH | Admitting team on call to chase and let metabolic/renal team |
| Feed/fluids plan (six hours before the anticipated start of the transplant surgery) | Needs IV fluids as by fasting protocol above prior to anaesthetics: 10% glucose + 0.45% sodium chloride at (maximum tolerated volume usually)/24hours – THIS IS TO RUN AT ALL TIME) | Any concerns, discuss with metabolic team |
| Metabolic monitoring | 4 hourly blood gas, ammonia, lactate | Discuss results with metabolic team |
| Immunosuppression | Methylprednisolone 600mg/m2 (max 1gram) before going to surgery.  Basiliximab 10mg (before surgery and on day 4 post op)  Tacrolimus 0.15mg/kg twice daily  MMF 600mg/m2 twice daily (timing of first dose to be discussed between renal and liver transplant teams) |  |

1. **Intraoperative management**

Please do not discontinue IV fluids. If blood glucose levels become elevated (above upper normal range), please start insulin and do not reduce dextrose concentration.

| **Metabolic monitoring** | **Hourly:**   - **Bloods:** Glucose***,*** Blood gas***,*** Lactate***,*** Ammonia U&Es   **4hrly :**  **Bloods:** Amylase, lipase, U+E, ketones  Patient is considered to be clinically unwell if:   - abnormal blood gas with ph < 7.3 and / or HCO3 < 16 and/or BE > - 4 and / or lactate > 4mmol/l - and / or ammonia > 100nmol - and / or amylase and / or lipase 2 x above normal - LFT 2 x above normal   **Recovery:**   - **Bloods:** Glucose, blood gas, lactate, ammonia, ketones, U+E, FBC, Coag, Amylase, Lipase, Plasma amino acids, MMA, acylcarnitine - **Urines:** MMA | Anaesthetist to liaise with metabolic/renal teams if concerns |
| --- | --- | --- |

1. **Management post operation in the first 24 hours**

| Bloods and urine monitoring | Two hourly creatinine, K+, urea, phosphate and FBC  X has native kidney output, so aim for at least >4ml/kg/hr urine as long as creatinine falling (if any concerns, contact renal consultant on call) | Inform renal team of all results once they are available |
| --- | --- | --- |
| Nutrition | ***Total daily volume and kcal intake to match daily maximum tolerated enteral feed volume as per pre-transplant maintenance feeds***  20% glucose/0.45% sodium chloride   - 90% of maximum volume tolerated / 24h - Plus added maintenance electrolytes as per renal team - 80% of daily calorie intake   TPN   - 10% of maximum volume tolerated / 24h - (0.5 x pre-transplant maintenance lipids) / kg / day - 20% of daily calorie intake   ***If possible, to re-start protein 24hours after transplantation-discuss with metabolic team:**  *****   - Re-start **protein** 24hours after transplantation (start with 0.5g protein/kg/day) - Post-Op Day 1: 0.5g/kg/day - Post-Op Day 2: 0.8g/kg/day - Post-Op Day 3: 1g/kg/day - BEFORE restarting protein check ammonia levels (should be < 80mmol/l) |  |
| Urinary catheter | To remain in situ until day 5 post op. If catheter falls out, do not attempt to change it (ureteric anastomosis into bladder can be damaged). Please inform renal transplant surgeon on call asap. |  |

1. **Key metabolic and renal contacts during admission**

**During hours (Monday-Friday 8am-6pm)**

Designated MMA Nephrologist

Specialist in Inherited Metabolic Medicine

Renal transplant surgeon

Renal and Liver transplant coordinators

Hepatologist

Metabolic advanced nurse practitioner

Metabolic dietician

**Out of hours (weekends and after 6pm Monday-Friday)**

Renal consultant on call

Metabolic consultant on call

***7. Additional:***

***1.*** *Monitor for Donor Specific Antibodies as follows:*

*• Recipients with anti-class I DSAs and delayed renal allograft function should have blood sent 2 x per week to Clinical transplant laboratory to check the DSA response whilst graft dysfunction is on-going as an inpatient.*

*• Recipients with anti-class II DSAs should have blood sent 2 x per week as an inpatient to Clinical transplant laboratory to check the DSA response.*

*• After discharge, send HLA antibody screens once a month*

***2.*** *Aim for tacrolimus levels of 10-12 in the first month, then 8-10 in the second month, and 5-7 thereafter.*
